# The Impact of Adherence and Instillation Proficiency of Topical Glaucoma Medications on Intraocular Pressure

**DOI:** 10.1155/2017/1683430

**Published:** 2017-09-14

**Authors:** Tesfay Mehari Atey, Workineh Shibeshi, Abeba T. Giorgis, Solomon Weldegebreal Asgedom

**Affiliations:** ^1^Clinical Pharmacy Unit, School of Pharmacy, College of Health Sciences, Mekelle University, Mekelle, Tigray, Ethiopia; ^2^Department of Pharmacology and Clinical Pharmacy, School of Pharmacy, College of Health Sciences, Addis Ababa University, Addis Ababa, Ethiopia; ^3^Department of Ophthalmology, School of Medicine, College of Health Sciences, Addis Ababa University, Addis Ababa, Ethiopia

## Abstract

**Background:**

The possible sequel of poorly controlled intraocular pressure (IOP) includes treatment failure, unnecessary medication use, and economic burden on patients with glaucoma.

**Objective:**

To assess the impact of adherence and instillation technique on IOP control.

**Methods:**

A cross-sectional study was conducted on 359 glaucoma patients in Menelik II Hospital from June 1 to July 31, 2015. After conducting a Q-Q analysis, multiple binary logistic analyses, linear regression analyses, and two-tailed paired t-test were conducted to compare IOP in the baseline versus current measurements.

**Results:**

Intraocular pressure was controlled in 59.6% of the patients and was relatively well controlled during the study period (mean (*M*) = 17.911 mmHg, standard deviation (*S*) = 0.323) compared to the baseline (*M* = 20.866 mmHg, *S* = 0.383, *t* (358) = −6.70, *p* < 0.0001). A unit increase in the administration technique score resulted in a 0.272 mmHg decrease in IOP (*p* = 0.03). Moreover, primary angle-closure glaucoma (adjusted odds ratio (AOR) = 0.347, 95% confidence interval (CI): 0.144–0.836) and two medications (AOR = 1.869, 95% CI: 1.259–9.379) were factors affecting IOP.

**Conclusion:**

Good instillation technique of the medications was correlated with a reduction in IOP. Consequently, regular assessment of the instillation technique and IOP should be done for better management of the disease.

## 1. Introduction

Glaucoma is a type of eye disorder resulting from optic neuropathy and leads to a progressive loss of retinal ganglion cell axons and ultimately irreversible blindness if left untreated [1–3]. It is the foremost cause of blindness among blacks [4] and the second leading cause of blindness globally next to cataract [5]. Worldwide, about 64 million people were affected by glaucoma in 2013 and this prevalence is expected to reach 76.0 and 111.8 million by 2020 and 2040, respectively [2]. Glaucoma inexplicably affects more Africans and Asians than whites [6] and it is considered as a public health problem in sub-Saharan Africa [7].

Because elevated intraocular pressure (IOP) is a major risk and causal factor for glaucoma [3], hence, hypotensive medications are prescribed as primary medications to control this pressure [8, 9]. Different studies proved that an elevated IOP hastens optic nerve head damage and waning of the visual field unless good adherence and appropriate instillation proficiency of topical glaucoma medications are strictly followed by the patients [10, 11].

During application of topical glaucoma medications into the eye, the administered dose could be lost through leakage and the punctum route. Approximately 80% of the administered drug could be removed from this route and go into the systemic circulation. Accordingly, the eyelid should be closed and the punctum route should be occluded in order to maximize the ocular bioavailability and to minimize the adverse systemic effects of these topically applied medications. Both these techniques serve to increase the therapeutic index of these eye drops and minimize dosage requirements [12, 13].

Previous studies showed that a large proportion of patients improperly administer their eye drops. For instance, a multicenter study from ten centers across Canada showed that over 50% of the patients were either nonadherent or demonstrated improper instillation proficiency [14]. A study conducted by Sleath et al. also found that 44% of the patients reported frequently missing the eye during attempted drop application [15]. A further study by Stone et al. also found that 17%–25% of patients were unable to effectively apply eye drop medications to their eye [16].

There are many factors that lead to poor control of IOP. Uncontrolled IOP could be due to poor adherence and/or incorrect instillation technique such as missing the eye during application. Possible sequel of poorly controlled IOP includes treatment failure, unnecessary use of additional medications, and economic burden on the patients [17–19]. The goal of every efficient antiglaucoma therapy is the attainment of target IOP. Target IOP is the level of IOP that is related to an insignificant likelihood of optic nerve damage and/or visual field loss. An association between the curve of IOP decrease and glaucoma progression is demonstrated in previous studies. In general, better protection from the loss of vision and visual field impairment in glaucoma patients could be achieved through reduced level of IOP [20]. Consequently, this study was done to assess the impact of adherence and instillation proficiency of topically applied medications on intraocular pressure among patients attending the glaucoma clinic of Menelik II Hospital.

## 2. Methods

The study was conducted at the glaucoma clinic of Menelik II Hospital (Addis Ababa, Ethiopia) using a cross-sectional study design. Patients, who attended the clinic from June 1, 2015, to July 31, 2015, were enrolled in the study to determine their level of intraocular pressure. Their medical records were reviewed for the type and severity of glaucoma and intraocular pressure in the baseline and current measurements. Baseline measurements and current measurements were extracted from the patients' record and were referring to the measurements of IOP during the first visit in the hospital and at the end of the data collection period, respectively. All glaucoma patients who were obtaining services at the clinic during the study period were considered as the study population. To select samples from the study population, a systematic random sampling technique was used in this study.

To estimate the minimum sample size required for the study, a single population estimating formula [21], accompanied by a conservative sample estimate (since there was no information from similar studies, past studies, or studies done on similar populations and no pilot study about the proportion was done), was used. The following points were taken into consideration during the sample size calculation: 95% confidence interval, 80% power of the study, 5% margin of error, 10% attrition, and 0.5 prevalence. Therefore, the number of study samples for this study was found to be 359. On average, 2110 patients were being served at the clinic per two months (statistics office of the hospital). Accordingly, the sampling interval (*k*) was calculated to be six (*k* = 2120/359 = 6). A starting number (i.e., two) was chosen randomly and blindly from number one to six so that patients were recruited in this study at every second interval from a list of six patients.

Besides the above methodological aspects, the following inclusion and exclusion criteria were applied in the study. Patients who were 18 years and older, diagnosed with glaucoma or ocular hypertension, were on eye drops for at least six months, had a regular follow-up, and had not undergone either laser or glaucoma surgery in the past three months were included in the study. Glaucoma patients with postoperative follow-up without having any medications and who were not willing to give informed written consent were excluded from the study.

An appropriate two-day training was given to three ophthalmic nurses before data collection. The data collectors had more than three years of work experience at the clinic, but neither recently nor currently working at the clinic during the study period. The data collection tool was pretested in 18 patients (5% of the sample size) to maximize the quality of data. Adherence to topical glaucoma medications and instillation technique were measured using the Morisky Medication Adherence Scale-8 [21–25] and a World Health Organization (WHO) recommended Eye Drop Instillation Technique [2, 9, 12, 26]. The IOP was measured in the hospital using a standardized and calibrated tonometry. Notwithstanding differences in the control of IOP among studies, a favorable strategy to achieve IOP control is 20% reduction from the initial IOP or below 18 mmHg in an advanced stage of glaucoma. In patients with initial glaucoma, 25% reduction from the initial IOP will slow down the disease progression by 45% [20]. Accordingly, in this study, an IOP was deemed to be controlled if there was more than 20% reduction for moderate and advanced glaucoma, and more than 25% in early glaucoma or the target IOP (10–21 mmHg) have been achieved.

Data were entered using Epi Info™ version 3.5.3 and analyzed using SPSS® version 21. Factors affecting controlled IOP were identified using multiple logistic regression. Likewise, multiple linear regression was done, after incorporating variables that were statistically significant at *p* < 0.2 during the bivariate analysis, to relate intraocular pressure with the level of adherence and instillation technique. After conducting Q-Q plots to determine the distribution normality of the IOP, a two-tailed paired *t*-test was also employed to assess the level of IOP control in the baseline versus the current measurements of IOP. Statistical significance for the aforementioned analyses was declared at *p* < 0.05.

## 3. Results

### 3.1. Sociodemographic and Clinical Characteristics

The response rate in this study was found to be 100%. Concerning the sociodemographic characteristic of the patients, more than two-thirds (69%) of the patients were males. The mean age of the participants was found to be about 61 ± 12.34 years ranging from 18 to 88 years. Furthermore, approximately one in three (32%) of the patients was retired. Despite the fact that a large proportion (90%) of the patients was living in urban areas, a lower educational level accounted for 64% of the patients. The sociodemographic data of these patients have been previously published [27, 28].

**Figure 1:**
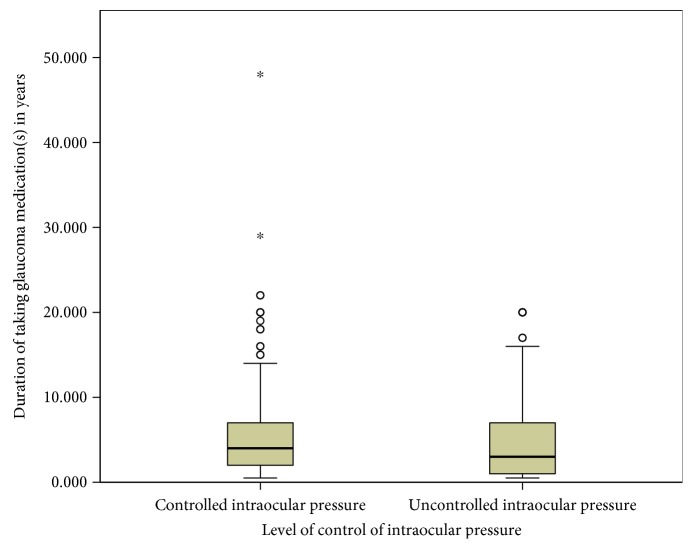
Level of intraocular pressure by the duration of taking glaucoma medications in Menelik II Hospital, 2015. ^*∗*^Outliers.

The mean duration of taking topical glaucoma medications for patients who had uncontrolled and controlled IOP were 4.92 years (standard error of mean (SE): ±0.40; 95% CI: 4.13–5.71; range: half a year to 20 years) and 5.61 years (SE: ±0.38; 95% CI: 4.87–6.35; range: half a year to 48 years) (Figure 1).

**Figure 2:**
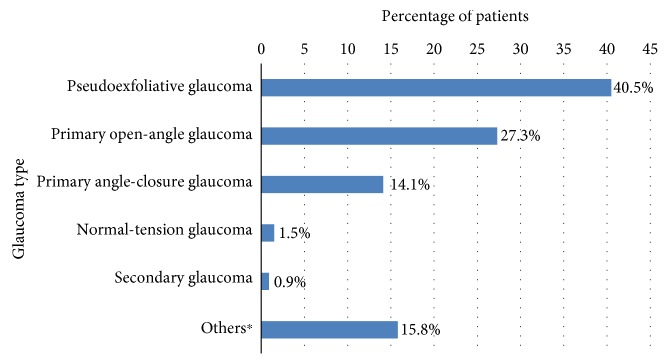
Profile of diagnosis of glaucoma among patients attending the glaucoma clinic of Menelik II Hospital, 2015. ^∗^Ocular hypertension, juvenile glaucoma.

The severity of glaucoma in these patients showed that advanced, moderate, and early glaucoma accounted for about 24%, 64%, and 12% of the patients, respectively. The most common type of glaucoma was pseudoexfoliative glaucoma, responsible for about 41% of the disease followed by primary open-angle glaucoma (27%) Figure 2.

In this study, based upon the international council of ophthalmology's classification for visual acuity [29], about 34%, 37%, and 32% of the patients were having (near) normal vision, low vision, and (near) blindness.

### 3.2. Eyelid Closure and Nasolacrimal Occlusion

**Figure 3:**
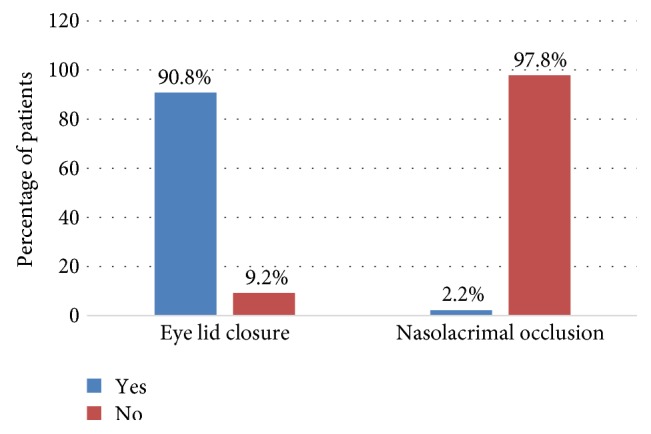
Percentage of practice of eyelid closure and nasolacrimal route occlusion among patients attending the glaucoma clinic of Menelik II Hospital, 2015.

Almost all of the study participants (98%) claimed that they were not occluding their nasolacrimal route during the application of glaucoma medications. In contrast, approximately 91% of the patients claimed the closure of their eyelid (Figure 3).

### 3.3. Level of Intraocular Pressure Control

**Figure 4:**
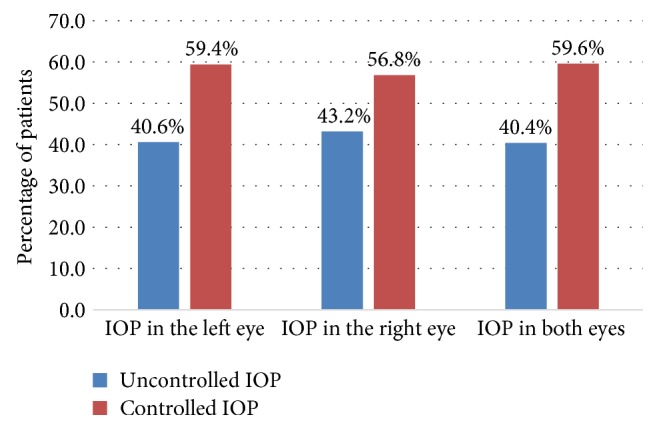
Comparison of the percentage of controlled intraocular pressure in the left, right, and both eyes among glaucoma patients in Menelik II Hospital, 2015.

The mean IOP, in mmHg, in the right eye and left eye was 17.8 (SD: ±7.7; range: 8 to 52) and 18.3 (SD: ±8.8; range: 6 to 61), respectively. For more than half of the patients, their IOP in the left eye (59%), right eye (57%), and both eyes (60%) were controlled using the glaucoma medications. The overall level of controlled IOP was found to be about 60% (Figure 4).

To test normality of the mean IOP of the patients for the purpose of linear regression analysis and paired *t*-test, a Q-Q plot was made. The Q-Q plots of IOP revealed that the pressure was almost normally distributed with a slight skewness to the left (−0.523 ± 0.129).

**Figure 5:**
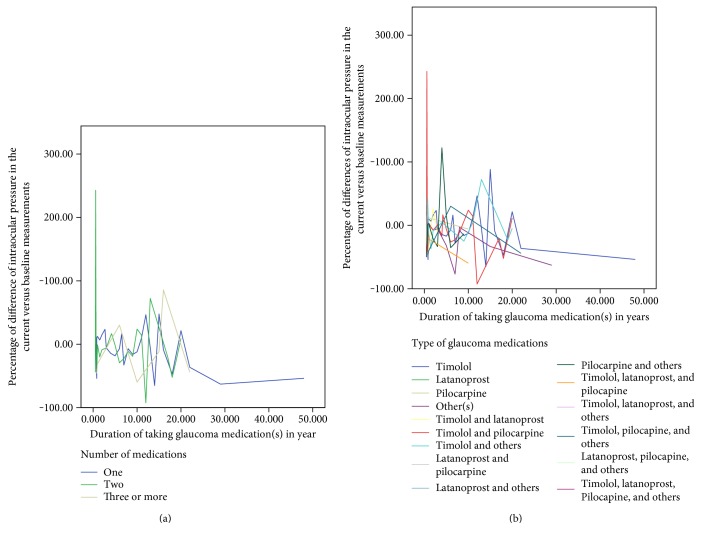
Relationship of intraocular pressure with the type and number of medications among glaucoma patients in Menelik II Hospital, 2015. (a) By the number of medications; and (b) by the type of medications.

Figure 5 shows a relationship of a percentage of difference IOP (reduction or increment) from the baseline in relation to the duration of taking glaucoma medication by a number of medications (panel a) and types of glaucoma medications (panel b). Generally, there was a greater reduction of intraocular pressure as the time of medications increased, as expected. A relatively slightly better IOP was controlled for patients taking timolol and pilocarpine compared to other medications (Figure 5).

Of the 113 patients who claimed to be highly adherent to their topical glaucoma medications, 57% of them had controlled IOP and the remaining (43%) had uncontrolled IOP. Likewise, among 62 patients who were appropriately instilling their topical glaucoma medications, 61% of them had controlled IOP compared to 39% of them whose IOP was not controlled.

Adherence status was found to be statistically associated with the instillation technique of topical glaucoma medications. Accordingly, the odds of appropriately instilling glaucoma medications were about 68% (crude odds ratio (COR) = 0.318, 95% CI: 0.174–0.579, *p* < 0.0001) and 76% (COR = 0.245, 95% CI: 0.096–0.621, *p* < 0.003) lower for patients with medium and low level of adherence, respectively, compared to those with high level of adherence (Table 1).

Regarding the association of the status of adherence with IOP, a unit increase in the score of nonadherence results in a 0.026 mmHg increase in IOP (*p* = 0.665). Concerning the administration technique, a unit increase in the score of administration technique results in a 0.272 mmHg decrease in IOP (*p* = 0.03) (Table 2).

The glaucoma patients had also a lower score of IOP during the study period (mean (*M*) = 17.911, standard error (*S*) = 0.323) compared to the baseline measurements (*M* = 20.866, *S* = 0.383, *t* (358) = −6.70, *p* < 0.0001).

### 3.4. Factors Associated with Intraocular Pressure

**Figure 6:**
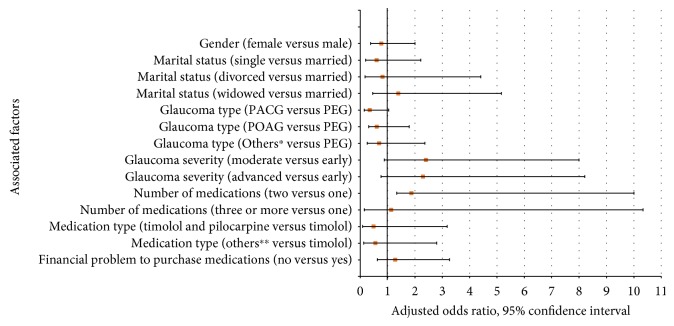
Factors associated with controlled intraocular pressure among patients attending the glaucoma clinic of Menelik II Hospital, 2015. The following factors were used in the logistic regression model: age, sex, marital status, ethnicity, educational level, residence, religion, occupation, monthly family income, type and severity of glaucoma, duration of the glaucoma in years, duration of taking medications in years, average follow-up period per year, the presence of previous surgery or laser treatment, major comorbidities, side effects of medications, acquisition of the medications (free of charge or not), financial problem to purchase the medications, the presence of other types of eye drops, adherence towards the medications, and instillation proficiency of the eye drops. The factors were assumed statistically significant at *p* < 0.05 and the end of the bar graph shows the odds ratio. PACG: primary angle-closure glaucoma; PEG: pseudoexfoliative glaucoma; POAG: primary open-angle glaucoma. ^∗^Secondary glaucoma, ocular hypertension, normal tension glaucoma, juvenile glaucoma ^∗∗^latanoprost; pilocarpine; timolol and latanoprost; timolol with other types of eye drops; pilocarpine with other types of eye drops; latanoprost with other types of eye drops; timolol, latanoprost, and pilocarpine; timolol and latanoprost with other types of eye drops; timolol and pilocarpine with other types of eye drops.

The list of factors associated with the IOP is summarized in Figure 6(). Accordingly, the glaucoma type and the number of glaucoma medications were found to be factors that were significantly associated with controlled IOP. Patients with primary angle-closure glaucoma were having 65% (adjusted odds ratio (AOR) = 0.347, 95% confidence interval (CI): 0.144–0.836, *p* < 0.018) lower odds of controlled IOP compared to patients with pseudoexfoliative glaucoma. Furthermore, the odds of having controlled IOP in patients who were taking two medications were almost twofold (AOR = 1.869, 95% CI: 1.259–9.379, *p* < 0.047) more compared to patients who were taking only one medication (Figure 6()).

## 4. Discussions

This study assessed the impact of glaucoma medications on the level of IOP control. In the previous publications, 42.6% of the patients were found to be adherent to their prescribed hypotensive agents [27] and the rate of the appropriate administration technique was also found to be 17.3% [28]. Despite the importance of assessing the adherence behavior towards the prescribed medications and administration technique of eye drops in glaucoma management, their effect on the treatment outcome of glaucoma, that is, intraocular pressure should be determined. Accordingly, for about 60% of the study participants, their IOP was controlled using the glaucoma medications. This finding might be substandard as substantiated by the findings that the majority (57%) of the patients were being nonadherent to their medications and most (83%) of the patients were not appropriately administering their topical glaucoma medications, according to the WHO guide. In this study, almost 60% of the study participants were neither adherent nor properly administered their eye drops, which was similar to studies that reported analogous rates in the USA [30], Greece [10], and Canada [14].

Besides the above findings, the present study also revealed that 77.2% of the study participants closed their eyes, but only 2.2% of them occluded their punctum route for at least two minutes during the administration procedure. Nevertheless, this result deviated from a study done in India that indicated a prevalence rate of about 29% of eyelid closure and 6% for punctum occlusion [18]. This difference might be instigated from discrepancies in patient education and awareness regarding instillation of eye drops and from variations in study methods. The practice of punctum occlusion was much poorer in the present study and almost all of the study participants admitted that they never occluded the punctum route. This poor practicing might be emanating from the poor patient education system and the unavailability of posters and brochures regarding instillation proficiency in the study center.

Among the patients who claimed to be adherent and who were appropriately instilling their medications, about two-thirds of them had controlled IOP. Being adherent and applying eye drops correctly maximize the intraocular concentration of the medications and minimize the systemic adverse effects. This could lead to a cumulative effect of better control of IOP. Furthermore, patients with high level of adherence were more likely to accurately administer their eye drops compared to patients with a low and medium level of adherence. This finding was attributable to the more cautious nature of the adherent patients in the correct instillation of their medications. This implied that adherence and instillation proficiencies are interconnected and poor practicing in instillation proficiency could jeopardize adherence and vice versa.

Another finding of this study also showed that a unit increase in a score of the nonadherence and in a score of the administration technique results in a 0.026 mmHg increase and a 0.272 mmHg decrease in the IOP, respectively. Improper instillation proficiency and poor adherence increase failure to deliver the desired drug to the eye and in turn lead to wasted medication. This, in turn, leads to poor IOP control and eventually augments frequent changes in the types of prescribed medications and more frequent hospital visits [31]. In contrast, enhancement of drug delivery, improvement in treatment effectiveness, and reduction of the number of patient visits to the hospital could be achieved through good adherence and proper instillation technique of the medications [31]. Thus, eye care providers and other stakeholders should give more emphasis on the proper education of adherence, instillation technique, and their effect on IOP control.

Glaucoma patients had better controlled IOP at the end of the study period compared to the baseline measurements. Despite the poor instillation proficiency and suboptimal adherence observed among the study participants, application of these medications results in the overall reduction of the IOP through their pharmacodynamic mechanism.

The number of medications and type of glaucoma were statistically associated with the level of IOP control. Accordingly, patients who were taking two glaucoma medications were more likely to have a controlled IOP compared to patients who were taking only one medication. Applying different medications with a different mechanism of action will effectively lower IOP more than a single medication. On the contrary, patients with primary angle-closure glaucoma had lower odds of controlled IOP compared to patients with pseudoexfoliative glaucoma and this might be attributable to the aggressive nature of the latter disease (i.e., pseudoexfoliative glaucoma) which tied to more attention and close follow-up for patients with this disease. On the other hand, the characteristics of the study participants might affect the nonexistence of a relationship observed among controlled IOP with sociodemographic factors, adherence level, and instillation proficiency. Demographic factors might have less influence on the level of IOP because of a longer duration of glaucoma (with a mean of 5.6 years) and a lengthy period of taking the topical glaucoma medications (with a mean of 5.4 years).

The present study has certain limitations. Primarily, the nature of the design, that is, cross-sectional, did not allow a longitudinal follow-up of the study participants to comprehensively identify the factors contributing for uncontrolled IOP. Secondly, two measurements, the baseline and current, were used to assess IOP control. The baseline measurement—which was assumed as the first measurement recorded during their first follow-up in the hospital—might not necessarily mean the actual baseline measurements as some of the patients might be referred from other eye care centers with medications. Besides this, variability in IOP measurements can occur as a function of instrumentation or even in patients' own diurnal variation. Thirdly, the value of controlled IOP depends on the pretreatment level of IOP and other factors. However, these factors were difficult to assess during the study period and hence, future studies should be done considering these factors. Lastly, self-reported adherence has been shown to be poorly predictive of adherence compared to objective measurements such as electronic monitoring. Therefore, objective measurements of adherence using drug concentrations and with longer assessment follow-up periods should be planned in future studies.

## 5. Conclusion

There was a substandard level of controlled intraocular pressure in the tertiary referral hospital. Good instillation technique of topical glaucoma medications is correlated with a reduction in intraocular pressure. Applying two topical glaucoma medications is found to be a contributing factor for having a controlled intraocular pressure. Consequently, regular assessment of the patients' instillation technique and intraocular pressure should be done for better management of the disease.

## Figures and Tables

**Table 1:** Association of medication adherence with instillation proficiency among patients attending the glaucoma clinic of Menelik II Hospital, 2015.

Adherence	Instillation proficiency, *n* (%)		COR (95% CI)	*p* value
	Inappropriate	Appropriate		
High adherence	79 (69.9)	34 (30.1)	ref	
Medium adherence	161 (88.0)	22 (12.0)	0.318 (0.174–0.579)	0.0001
Low adherence	57 (90.5)	6 (9.5)	0.245 (0.096–0.621)	0.003

CI: confidence interval; COR: crude odds ratio.

**Table 2:** Association of intraocular pressure with adherence and administration technique among patients attending the glaucoma clinic of Menelik II Hospital, 2015.

Variable	Beta estimate (SE)	CI (*p* value)
Adherence	0.026 (0.325)	−0.613 to 0.665 (0.936)
Administration technique	−0.272 (0.214)	−0.692 to −0.149 (0.03)

CI: confidence interval; SE: standard error of mean.

## Abbreviations

AOR: Adjusted odds ratio
CI: Confidence interval
COR: Crude odds ratio
df: Degree of freedom
IOP: Intraocular pressure
M: Mean
mmHg: Millimeters of mercury
SD: Standard deviation
SE (*S*): Standard error of mean.

## Additional Points

*Supporting Information*. The datasets supporting the conclusions of the study are available publicly in server of Addis Ababa University. Any additional data will be available on request.
